# Zebrafish-based identification of the antiseizure nucleoside inosine from the marine diatom *Skeletonema marinoi*

**DOI:** 10.1371/journal.pone.0196195

**Published:** 2018-04-24

**Authors:** Théo Brillatz, Chiara Lauritano, Maxime Jacmin, Supitcha Khamma, Laurence Marcourt, Davide Righi, Giovanna Romano, Francesco Esposito, Adrianna Ianora, Emerson F. Queiroz, Jean-Luc Wolfender, Alexander D. Crawford

**Affiliations:** 1 School of Pharmaceutical Sciences, EPGL, University of Geneva, University of Lausanne, Genève, Switzerland; 2 Integrative Marine Ecology Department, Stazione Zoologica Anton Dohrn, Napoli, Italy; 3 Luxembourg Centre for Systems Biomedicine, Université du Luxembourg, Belvaux, Luxembourg; 4 Theracule S.á r.l., Belval, Luxembourg; 5 Faculty of Veterinary Medicine, Norwegian University of Life Sciences, Oslo, Norway; University of Modena and Reggio Emilia, ITALY

## Abstract

With the goal of identifying neuroactive secondary metabolites from microalgae, a microscale *in vivo* zebrafish bioassay for antiseizure activity was used to evaluate bioactivities of the diatom *Skeletonema marinoi*, which was recently revealed as being a promising source of drug-like small molecules. A freeze-dried culture of *S*. *marinoi* was extracted by solvents with increasing polarities (hexane, dichloromethane, methanol and water) and these extracts were screened for anticonvulsant activity using a larval zebrafish epilepsy model with seizures induced by the GABA_A_ antagonist pentylenetetrazole. The methanolic extract of *S*. *marinoi* exhibited significant anticonvulsant activity and was chosen for bioassay-guided fractionation, which associated the bioactivity with minor constituents. The key anticonvulsant constituent was identified as the nucleoside inosine, a well-known adenosine receptor agonist with previously reported antiseizure activities in mice and rat epilepsy models, but not reported to date as a bioactive constituent of microalgae. In addition, a UHPLC-HRMS metabolite profiling was used for dereplication of the other constituents of *S*. *marinoi*. Structures of the isolated compounds were elucidated by nuclear magnetic resonance and high-resolution spectrometry. These results highlight the potential of zebrafish-based screening and bioassay-guided fractionation to identify neuroactive marine natural products.

## Introduction

Epilepsy is one of the most common neurological disorders, and its unpredictable seizures affect approximately 50 million people of all ages worldwide [1]. Despite the availability of over 25 approved anti-epileptic drugs (AEDs), a third of treated epileptic patients experience pharmaco-resistance or serious side effects to these medications [2, 3]. There is therefore an urgent need to search for new compounds to treat this disease. An attractive source of novel drug leads for epilepsy are medicinal plants and other natural sources [4].

Here we evaluate extracts from diatoms for potential anticonvulsant activity using an epilepsy bioassay based on zebrafish larvae (*Danio rerio*) [5]. Advantages of this vertebrate model include their small size, rapid development and homology to humans, as well as their suitability for the rapid *in vivo* screening of small molecules for bioactivity [6]. Zebrafish are now well established as a screening platform to rapidly assess the anticonvulsant activity of compounds, using both pharmacological and genetic seizure models. For the seizure assay used for this study, zebrafish larvae were treated with the GABA_A_ antagonist pentylenetetrazole (PTZ), which induces epileptic seizures that can be quantified both by behavioral analysis and by electrophysiological analysis.

We have recently established zebrafish as a platform for biodiscovery [6–8]. To date, by using zebrafish seizure models for *in vivo* bioassay-guided fractionation, we have isolated several anticonvulsant secondary metabolites from medicinal plant extracts, including bisabolene sesquiterpenoids from the Ayurvedic medicinal plant *Curcuma longa*, tanshinones from the Chinese medicinal plant *Salvia miltiorrhiza* and spirostane glycosides from the Philippine medicinal plant *Solanum torvum* [9–11]. We have also recently highlighted the potential of zebrafish and other *in vivo* models, such as *Caenorhabditis elegans* and *Drosophila*, for marine biodiscovery [12].

Diatoms, with over 100,000 species, constitute one of the major components of marine phytoplankton, comprising up to 40% of annual productivity at sea and representing 25% of global carbon-fixation [13]. Different studies have shown that diatoms are excellent sources of pigments, lipids, carotenoids, ω-3 fatty acids and bioactive compounds [14–19]. Some diatom species have notably demonstrated teratogenic activity against their predators, but also anti-proliferative activity against both bacteria and cancer cell lines [20–23]. Diatom extracts have, however, only rarely been investigated for potential neuroprotective and other CNS-relevant bioactivities. Interestingly, the cyclic imine toxin 13-desmethyl spirolide C, a polyketide that targets nicotine receptors, has recently been identified in the dinoflagellate *Alexandrium ostenfeldii* for its potential against Alzheimer disease [24].

The diatom *Skeletonema marinoi* is generally considered as a model species in chemical-ecology studies [25–27], due to its well-known antiproliferative activity on its predators, as well as antimicrobial, anticancer and antioxidant activities [28]. Considering the demonstrated biotechnological and ecological relevance of this species, several studies have been performed or are in progress in order to screen other possible bioactivities and biotechnological applications, as well as to explore ecological aspects [29, 30]. The complete mitochondrial genome of *S*. *marinoi* has recently been published [31] and the full genome is currently in progress (http://cemeb.science.gu.se/research/target-species-imago/skeletonema-marinoi).

Within the context of a large-scale *in vivo* screening of secondary metabolites from marine microorganisms to identify neuroactive marine natural products [32, 33], extracts of diatoms and other microalgae were screened for neuroactivity using a zebrafish-based behavioral fingerprinting assay [34]. For the antiseizure activity, a zebrafish epilepsy assay was performed based on the proconvulsant PTZ, a GABA_A_ receptor antagonist [5]. Our subsequent analysis of the methanolic extract of *S*. *marinoi*, described here, revealed significant anticonvulsant activity. To our knowledge, this is the first report of this type of bioactivity for diatoms. Here we show that the anticonvulsant activity of the diatom *Skeletonema marinoi* is due to the production of the nucleoside inosine, a well-known antiepileptic compound.

## Results

### Anticonvulsant activity of the *S*. *marinoi* extracts

**Fig 1:**
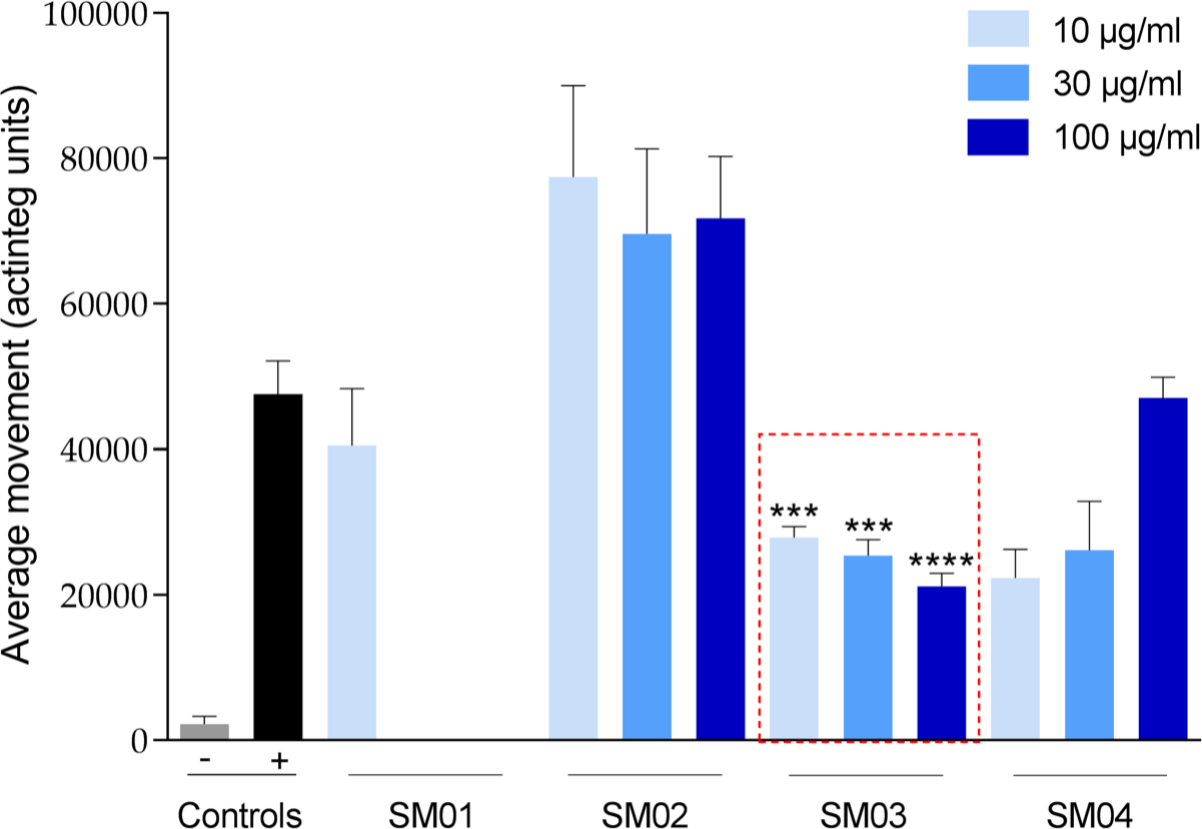
Larval locomotor activity (expressed in actinteg units) of *S*. *marinoi* extracts tested on zebrafish larvae. Hexanic extract (SM01), dichloromethane extract (SM02), methanolic extract (SM03) and aqueous extract (SM04) were tested at 10, 30 and 100 μg/mL concentrations. PTZ was used at 20 mM as the proconvulsant agent. Controls are described as “-” for the vehicle control and “+” for the positive control. Data are represented as Actinteg mean ± SEM (n ≥ 5). Statistical analysis was performed by one-way ANOVA with Dunett’s test to compare samples with positive control, with *P* values of <0.001 (***), <0.0001 (****).

The marine diatom *S*. *marinoi* was tested for potential anticonvulsant activity using the PTZ epilepsy zebrafish model, revealing a reduction of PTZ-induced movement by 62% at 3 μg/mL. In order to better understand the type of compounds responsible for this significant anticonvulsant activity, the freeze-dried culture of *S*. *marinoi* was extracted by solvents with increasing polarities (hexane, dichloromethane, methanol and distilled water). The anticonvulsant activity of the crude extracts was then submitted again to the zebrafish epilepsy assay (Fig 1). The methanol extract (SM03) revealed the strongest anticonvulsant activity, decreasing PTZ-induced locomotor activity by up to 55% at all the concentrations tested (p<0.001 for both 10 μg/mL and 30 μg/mL and p<0.0001 for 100 μg/mL). The water extract (SM04) also decreased locomotor activity at 10 and 30 μg/mL, while at 100 μg/mL it induced toxicity with an augmentation of fish movements. In contrast, the dichloromethane extract (SM02) was not active and exhibited a significant increase in locomotor activity at the three concentrations tested. The hexane extract (SM01), on the other hand, was toxic for the larvae at 30 and 100 μg/mL.

The most active extract, SM03, did not cause any sign of locomotor impairments in the zebrafish larvae at the maximum tolerated concentration (MTC). For this, an independent and wider series of dilutions were evaluated, from 10 to 300 μg/mL. Anticonvulsant activity was confirmed at 10, 30 and 100 μg/mL. The bioactivity at 200 μg/mL was similar to that observed at 100 μg/mL, while a concentration of 300 μg/mL was found to be toxic for the zebrafish larvae. Due to the clear anticonvulsant activity exhibited by SM03, this extract was submitted to a metabolite profiling and a bioassay-guided isolation in order to identify the compounds responsible for this bioactivity.

### Metabolite profiling

In order to obtain an initial insight of the composition of *S*. *marinoi*, the methanolic extract was submitted to a metabolite profiling by UHPLC-TOF-HRMS. The dereplication gave molecular formula determinations based on HRMS^1^ spectra and was cross searched with a custom database built from the Dictionary of Natural Products (DVD version 25:2) [35], with a selection restricted to natural products from cyanobacteria, green and red algae (total of 4043 compounds). This allowed the putative identification of 11 secondary metabolites and to the best of our knowledge, these compounds are here described for the first time in *S*. *marinoi* (Table 1). Compound (**3**) *m/z* 247.1707 [M+H]^-^, C_16_H_24_O_2_ (Δppm = 0.5) was dereplicated as 10-phenyldecanoic acid [36]; (**4**) *m/z* 489.3075 [M+H]^-^, C_25_H_46_O_9_ (Δppm = 3.0), as a derivative of glycerol 1-(9-hexadecenoate) [37]; (**5**) *m/z* 649.3427 [M+H]^-^, C_34_H_46_N_6_O_7_ (Δppm = 10.8), as aeruginosin KT650 [38]; (**6**) *m/z* 249.1869 [M+H]^-^, C_16_H_26_O_2_ (Δppm = 3.7), as 8,11,14-hexadecatrienoic acid or 6,10,14-hexadecatrienoic acid [39]; (**7**) *m/z* 552.2343 [M+H]^-^, C_26_H_47_IO_4_ (Δppm = 1.5), as a iodolactone derivative [40]; (**8**) *m/z* 351.2533 [M+H]^+^, C_21_H_34_O_4_ (Δppm = 2.3), as 9-*O*-methylconstanolactone B [41]; (**9**) *m/z* 329.2668 [M+H]^+^, C_19_H_36_O_4_ (Δppm = 2.0), as ceratodictyol A or B [42]; (**10***) m/z* 609.2734 [M+H]^+^, C_35_H_36_N_4_O_6_ (Δppm = 4.5), as 10-hydroxyphaeophorbide A [43]; (**11**) *m/z* 313.2725 [M+H]^+^, C_19_H_36_O_3_ (Δppm = 3.0), as tetrahydro-2-(1-hydroxy-9-nonenyl)-5-pentyl-3-furanol [44]; (**12**) *m/z* 285.2411 [M+H]^+^, C_17_H_32_O_3_ (Δppm = 4.7), as tanikolide [45]; (**13**) *m/z* 303.2532 [M+H]^+^, C_17_H_34_O_4_ (Δppm = 0.6), as secotanikolide [46]. Isomers were found for (**7**) at a retention time of 15.30 and 15.70 min, (**9**) at 17.45 and 18.01 min, (**10**) at 24.73 and 25.49 min, (**11**) at 21.14 and 21.94 min. The negative ion (NI) ESI-HRMS spectrum of (**1**) displayed an ion at *m/z* 267.0726 [M+H], which is in agreement with the molecular formula C_10_H_12_N_4_O_5_ (Δppm = -3.2), while the positive ion (PI) ESI-HRMS spectrum of (**2**), *m/z* 197.1134 [M+H]^+^, C_11_H_16_O_3_ (Δppm = -18.6). In parallel with the metabolite profiling, this extract was submitted to a one-step preparative chromatography separation after vacuum liquid chromatography (VLC) enrichment to determine if the activity could be linked to one of these metabolites.

**Table 1:** Metabolite profiling of SM03 by UHPLC-HRMS. This table indicates putative molecular formula based on HRMS^1^ spectra, cross searched with a custom database built from the Dictionary of Natural Products, with a selection restricted to natural products occurring in cyanobacteria, green and red algae.

RT (min)	Isolated as	Dereplicated as	Molecular formula	Organism	Ionization mode (*m/z*)	Error (ppm)	Isotopic pattern score (%)	CRC number	Reference
1.12	Inosine	Inosine (**1**)	C_10_H_12_N_4_O_5_	Diatom	[M+H]^-^	3.2			[47]
6.02	Loliolide	Loliolide (**2**)	C_11_H_16_O_3_	Diatom	[M+H]^+^				[48]
15.30		Iodolactone derivative (**7**)	C_26_H_47_IO_4_	Red algae	[M+H]^-^	3.0	90.2	FOW42	[40]
15.65		10-Phenyldecanoic acid (**3**)	C_16_H_24_O_2_	Green algae	[M+H]^-^	0.5	91.4	HPM75 / DFC94	[36]
15.98		Aeruginosin KT650 (**5**)	C_34_H_46_N_6_O_7_	Cyanobacteria	[M+H]^-^	10.8	94.7	QQR75	[38]
16.38		8,11,14-Hexadecatrienoic acid or 6,10,14-Hexadecatrienoic acid (**6**)	C_16_H_26_O_2_	Green algae	[M+H]^-^	3.7	97.8	MSG22 / MTB49	[39]
16.52		9-O-Methylconstanolactone B (**8**)	C_21_H_34_O_4_	Red algae	[M+H]^+^	2.3	92.5	NKK80	[41]
17.28		Tanikolide (**12**)	C_17_H_32_O_3_	Cyanobacteria	[M+H]^+^	4.7	95.7	HCY62	[45]
17.45		Ceratodictyol A or Ceratodictyol B (**9**)	C_19_H_36_O_4_	Red algae (associated to a sponge)	[M+H]^+^	2.0	97.9	QFY97 / QFY99	[42]
17.79		Secotanikolide (**13**)	C_17_H_34_O_4_	Cyanobacteria	[M+H]^+^	0.4	94.5	PYQ90	[46]
18.01		Ceratodictyol A or Ceratodictyol B (**9**)	C_19_H_36_O_4_	Red algae (associated to a sponge)	[M+H]^+^	2.0	97.7	QFY97 / QFY99	[42]
19.79		Glycerol 1-(9-hexadecenoate) derivative (**4**)	C_25_H_46_O_9_	Cyanobacteria	[M+H]-	3.0	93.6	CDO10	[37]
21.94		Tetrahydro-2-(1-hydroxy-9-nonenyl)-5-pentyl-3-furanol (**11**)	C_19_H_36_O_3_	Brown algae	[M+H]^+^	3.0	93.0	FOB08	[44]
25.49		10-Hydroxyphaeophorbide A (**10**)	C_35_H_36_N_4_O_6_	Green algae (*Chlorella sp*.*)*	[M+H]^+^	4.5	95.9	BOO46	[43]

### Bioassay guided fractionation

**Fig 2:**
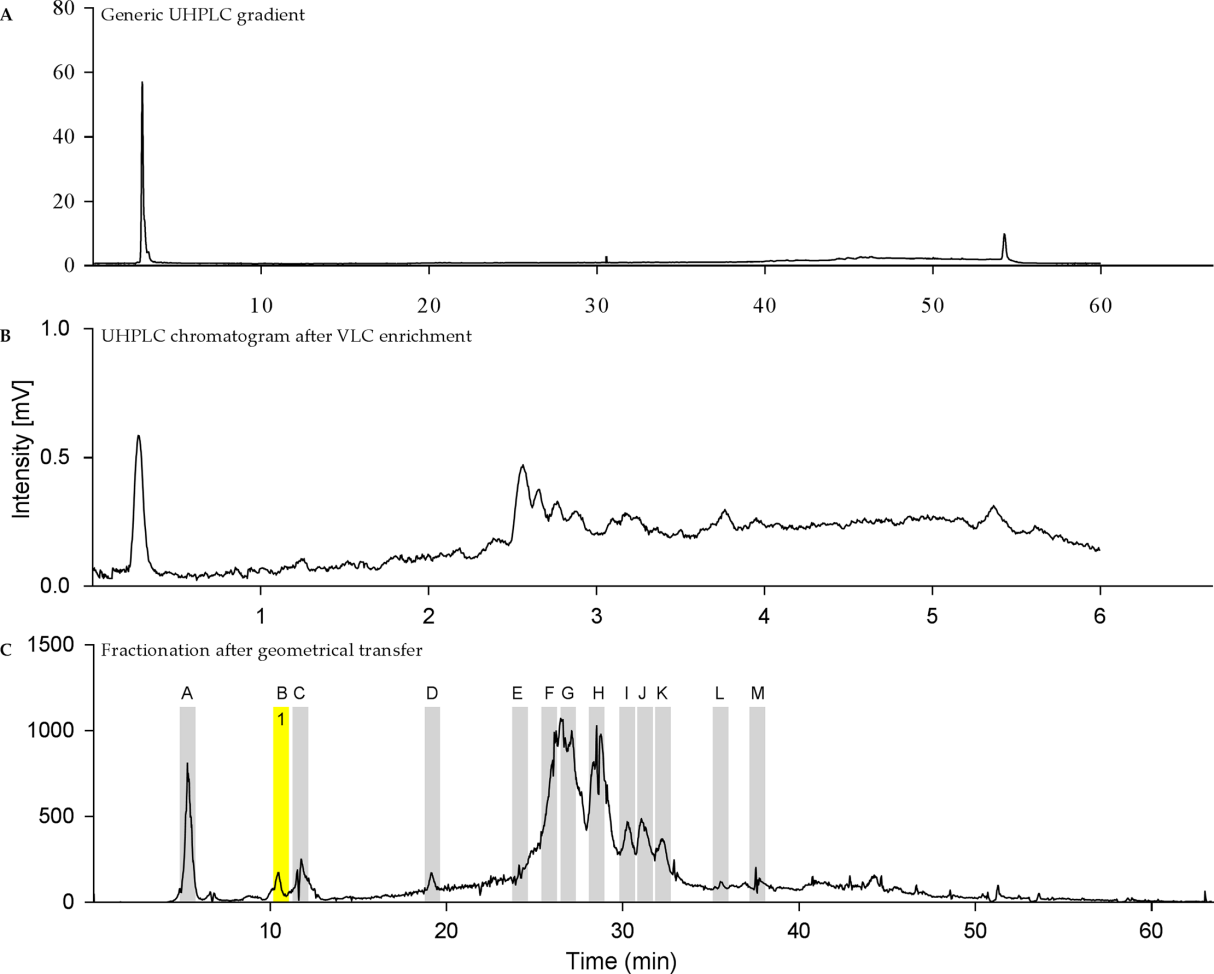
ELSD chromatograms for the methanolic extract before and after enrichment, and its fractionation by preparative chromatography. (A) UHPLC chromatogram of the crude methanolic extract exhibiting mainly unretained polar metabolites. (B) UHPLC metabolite profiling after enrichment by VLC to remove very polar residues. (C) Preparative chromatography for the fractionation of the enriched extract highlighting the isolation of constituent **1**.

**Fig 3:**
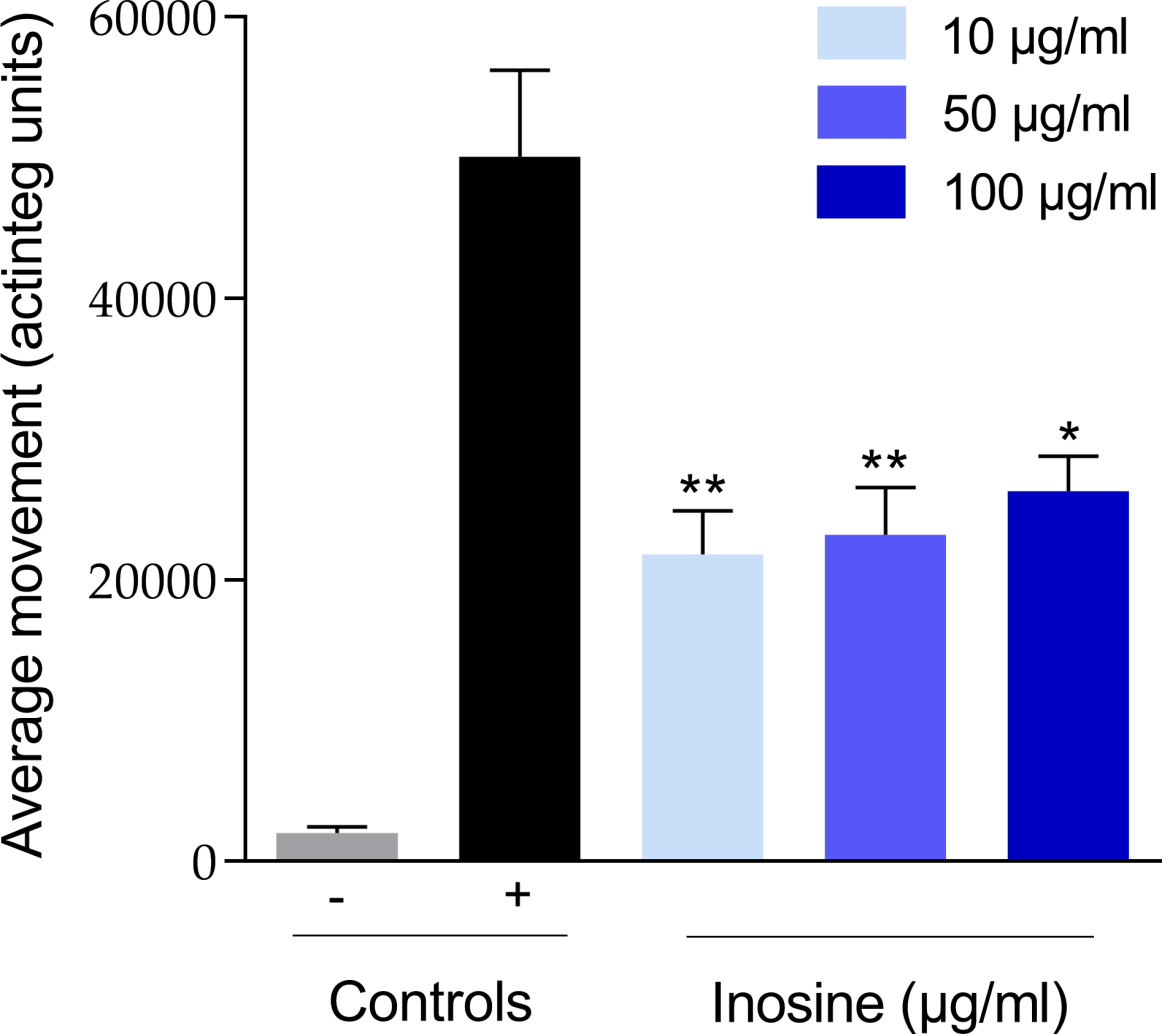
Effects of inosine on larval locomotor activity after 30 min tracking period (expressed in actinteg). Inosine (1) was tested at 10, 50 and 100 μg/mL. PTZ was used at 20 mM as proconvulsant agent. Controls are indicated as “-” for the vehicle control and “+” for the positive control. Data are represented as Actinteg mean ± SEM (n ≥ 3). Statistical analysis was performed by one-way ANOVA with Dunett’s test to compare samples with positive control, with *P* values of <0.05 (*), <0.01 (**).

The methanolic extract SM03 was first subjected to VLC in order to remove very polar residues (Fig 2A and 2B), and then fractionated by preparative chromatography. For an efficient fractionation, the preparative chromatographic conditions were determined based on a gradient transfer from optimized high-performance liquid chromatography (HPLC) conditions using the same stationary phase (Fig 2C). The fractionation was monitored by UV and evaporative light scattering (ELSD) detectors and led to 158 fractions. Only the 13 richest regions of the chromatogram possessing ELSD peaks (Fig 2C) were selected for evaluation with the zebrafish epilepsy assay, namely fraction A to M (S1 Fig.). A second screening was performed for these fractions to also evaluate the MTC and only fraction B showed a significant decrease of PTZ-induced locomotor activity. In order to confirm its activity, it was evaluated again at different concentrations (10, 50 and 100 μg/mL) and the results were significant for all of the concentrations tested (p<0.01 for 10 and 50 μg/mL, and p<0.05 for 100 μg/mL), with a 56% reduction of the PTZ-induced seizures at 10 μg/mL (Fig 3).

The active fraction was associated with peak (**1**) at a retention time of 10.3 min in the ELSD preparative chromatography trace of the enriched extract (Fig 2C). Analysis of fraction B by UHPLC-HRMS for the metabolite profiling (see 2.2) revealed that the fraction contained a single constituent. The corresponding molecular formula C_10_H_12_N_4_O_5_ was searched against entries in the DNP libraries for compounds occurring in cyanobacteria, green and red algae and could not be dereplicated. Nuclear magnetic resonance (NMR) allowed the unambiguous identification of the active compound as inosine (**1**) (S1 Table, S2–S6 Figs). In order to evaluate the quantity of inosine in *S*. *marinoi* freeze dried pellets, this compound was quantified by NMR indicating a significant amount of inosine (0.19% w/w) [49, 50]. Together with this active compound, the fractionation of SM03 revealed another pure constituent fully characterized as loliolide reported for the first time in *S*. *marinoi*. Loliolide was not active in the zebrafish epilepsy model.

## Discussion

The marine environment represents a significant reservoir for the discovery of new drugs because of its vast chemical and biological diversity. Compounds with neuroprotective and anticonvulsant activities have been found in both marine macro- and microorganisms [26, 51–55]. Examples of marine natural products with anticonvulsant activities are ceramides from the Red Sea sponge *Negombata corticata* [51], a nonsteroidal sesterterpene manoalide from the marine sponge *Luffariella variabilis* [53], semi-purified fractions from the sponge *Spongia officinalis* [52], conantokin-R from the venoms of marine cone snails [54] and a farnesylacetone epoxide from the brown macroalga *Cystophora moniliformis* [55]. Regarding microalgae, a cyclic imine toxin, 13-desmethyl spirolide C from the dinoflagellate *Alexandrium ostenfeldii*, showed potential activity against Alzheimer disease [26], but to date no CNS-active compounds have been reported from diatoms.

Here, we report the anticonvulsant activity of secondary metabolite extracts of the marine diatom *Skeletonema marinoi* and the identification of an active anticonvulsant constituent, inosine. Solvents with increasing polarities (hexane, dichloromethane, methanol and water) were used to obtain extracts with different metabolite profiles. The methanolic extract, generally used for the extraction of polar compounds [56], showed the strongest significant anticonvulsant activity, reducing seizure-related movement activity in PTZ-treated zebrafish larvae by 55% at 100μg/ml. This PTZ seizure model was then used for zebrafish bioassay-guided fractionation [5, 11] of the diatom extract, resulting in the isolation of inosine, which inhibited PTZ-induced seizures by 56% at 10 μg/ml [35, 57, 58].

Inosine, as well as adenosine and some other non-adenosine nucleosides (e.g. guanosine and uridine), are known to be modulatory molecules in the central nervous system, regulating various physiological and pathophysiological processes in the brain, such as sleep and epileptic seizures [59]. Indeed, Kovács and colleagues highlighted that cerebral spinal fluid levels of purine nucleosides (adenosine, guanosine and inosine) are altered after a single seizure episode and suggested that purine nucleosides have an important role in terminating seizures [60]. In addition, these studies reported that inosine exerts its anticonvulsant effects by acting on benzodiazepine binding sites in GABA_A_ receptors. Consequently, inosine was shown to increase the latency of PTZ-induced locomotor activity in mice and rat models [61]. Finally, inosine reached phase III clinical trials (USA, NCT02642393) for the study of urate elevation in Parkinson’s disease and phase II for the treatment of ALS (USA, NCT02288091) [62, 63]. In this work, we could demonstrate the validity of the PTZ-treated zebrafish larvae model for screening extracts from marine origin and for the efficient identification of their anticonvulsant metabolites.

## Materials and methods

### General experimental procedure

NMR ^1^H and ^13^C spectroscopic data were recorded on a Varian (Palo Alto, CA, USA) Unity Inova 500 MHz spectrometer. Chemical shifts (δ) are measured in parts per million (ppm) using the CD_3_OD signal as internal standard for all spectra (δ_H_ 3.31; δ_C_ 49.0), and coupling constants (*J*) are reported in Hz. Complete assignment was performed based on two-dimensional experiments (COSY, NOESY, HSQC and HMBC). High resolution mass spectrometry data were obtained on a Micromass LCT Premier Time-Of-Flight (TOF) mass spectrometer from Waters with an electrospray ionization (ESI) interface (Waters, Milford, MA). Reverse phase analyses were first performed on a high-performance liquid chromatography (HPLC) Agilent 1260 Infinity II LC system consisting of a degasser, a mixing pump, an autosampler, and a diode array detector (PDA) (Agilent Technologies, Santa Clara, USA) connected to an evaporative light scattering detector (ELSD) Sedex LT-ELSD 85 (Sedere, Oliver, France). Fractionation was performed on a Puriflash 4250 preparative chromatographic system (Interchim, Montluçon Cedex, France) equipped with a quaternary pump, a photodiode array detector, and a fraction collector.

### Cell culturing and harvesting

Seventeen different replicates of the centric diatom *S*. *marinoi* (clone FE6 from the Stazione Zoologica culture collection previously identified with both microscopy and 18S ribosomal sequencing) were cultured in Guillard’s f/2 medium in ten-liter polycarbonate carboys. *S*. *marinoi* cultures were constantly bubbled with air filtered through 0.2 μm membrane filters and kept in a climate chamber at 19°C and a 12:12 hours light:dark cycle (100 μmol photons m^−2^.s^−1^) [64]. Initial cell concentrations were about 5000 cells/mL; culture growth was monitored daily by fixing 1 ml of culture with one drop of Lugol (final concentration of about 2%) and counting cells in a Bürker counting chamber under an Axioskop 2 microscope (20×) (Carl Zeiss GmbH, Jena, Germany). At the end of the stationary phase, cultures were centrifuged for 15 min at 4°C at 3900 g using a cooled centrifuge with a swing-out rotor (DR 15P, Braun Biotechnology International, Allentown, PA, USA). The supernatant was discarded and pellets were freeze-dried and kept at −80°C until chemical extraction.

### Preparation of extracts and fractionation

Freeze dried pellets of *S*. *marinoi* were pooled together and ground under liquid nitrogen to avoid degradation. A successive extraction by solvents with increasing polarities (hexane, dichloromethane, methanol and water respectively) was performed under agitation on the *S*. *marinoi* powder (9.17 g). Each extract was concentrated under vacuum (Büchi Rotavapor) and then dried with a nitrogen flow until complete evaporation of solvent. The extractions afforded 0.50 g of hexane (5.45%), 0.48 g of dichloromethane (5.23%), 4.16 g of methanol (45.37%) and 2.02 g of aqueous (22.03%) extracts. Samples were named as hexane (SM01), dichloromethane (SM02), methanol (SM03) and aqueous (SM04) extracts. The methanolic extract (4.16 g) was subjected to a VLC in order to remove very polar compounds. A 250 mL sintered-glass Büchner funnel attached to a vacuum line was packed with a C18 reverse phase LiChroprep^®^ 40–63 μm (Lobar^®^ Merck, Darmstadt, Germany). The stationary phase was first conditioned with methanol (4 x 250 mL) followed by distilled water (4 x 250 mL). The dry load was prepared by mixing 4.16 g of the extract with 5 g of C18 reverse phase LiChroprep^®^ 40–63 μm (Lobar^®^ Merck, Darmstadt, Germany). The mixture was introduced from the top of the VLC by dry load for uniform loading. Elution of the sample was performed using water (4 x 250 mL) followed by methanol (4 x 250 mL) and was washed with ethyl acetate (4 x 250 mL). This yielded the following VLC fractions: SM03_VLC_H_2_O (1.50 g), SM03_VLC_MeOH (0.53 g) and SM03_VLC_EtOAc (0.46 g). The VLC methanolic fraction was then separated by preparative chromatography.

### HPLC-PDA-ELSD analysis

Extracts and fractions were analyzed by HPLC with UV and ELSD detection on a X-Bridge C18 column (250 x 4.6 mm i.d., 5 μm; Waters, Milford, MA, USA) using a mobile phase consisting of water (A) and methanol (B) containing both 0.1% formic acid; separation was performed with a linear gradient from 5 to 95% of B in 40 min; Injection volume was set at 10 μL, the flow rate was fixed at 1 mL/min. The samples were diluted in methanol at 5 mg/mL. ELSD conditions: 55°C, 3.1 bar N_2_ and gain 8.

### UHPLC-TOF-HRMS analysis

Ultra high-performance liquid chromatography coupled to Time of Flight (UHPLC-TOF) high resolution mass spectrometry was used to monitor extracts, fractions and pure compounds. The instrument used was a Waters^®^ Acquity UPLC system coupled to a Waters^®^ Micromass LCT Premier Time-Of-Flight (TOF) mass spectrometer (Waters, Milford MA, USA) equipped with an electrospray interface (ESI). ESI conditions were as follow: capillary voltage: 2400 V; cone voltage: 40 V; source temperature: 120°C; desolvation temperature: 300°C; cone gas flow 20 L/h; desolvation gas flow 600 L/h; MCP detector voltage: 2.4 kV; positive mode: 2.8 kV and negative mode: 2.4 kV. Detection was performed in positive ion mode (PI) and in negative ion mode (NI) with a m/z range of 100–1300 Da and a scan time of 0.3 sec in the centroid mode. The mass spectrometer was calibrated using sodium formate, and leucine encephalin. UHPLC analysis were performed on a Acquity UPLC System (Waters, Milford MA, USA) using a Acquity BEH C18 column (1 x 50 mm; 1.7 μm; Waters, Milford, MA, USA). The temperatures in the auto sampler and in the column oven were fixed at 10 and 40°C, respectively. The mobile phase consisted of water (A) and acetonitrile (B) containing both 0.1% formic acid; separation was performed with a linear gradient from 5% to 95% of B in 4 min followed by a 0.8 min isocratic step at 95% of B and then 1.2 min isocratic step at 5% of B for column reconditioning. Injection volume was set at 1 μL, the flow rate was fixed at 0.46 mL/min. The samples were diluted in methanol at 100 μg/mL.

### Fractionation of the VLC methanolic fraction

The optimized analytical HPLC conditions were geometrically transferred for fractionation on a Puriflash 4250 preparative chromatographic system (Interchim, Montluçon Cedex, France) equipped with a quaternary pump, a UV and a ELSD detectors, and a fraction collector [65]. The separation was performed using a HQ C18 column (PF-C18/120 g, 15 μm; Interchim, Montluçon Cedex, France) with water (A) and methanol (B) containing both 0.1% formic acid as mobile phase. The separation was achieved using a step gradient from 5 to 85% of B in 21 min and then 85 to 100% of B in 19 min. The flow rate was fixed at 26 mL/min. The UV detection in the scan mode (200–600 nm) and ELSD (35°C, gain 8, 3.5 bar, 1 mL/min). The separation afforded 158 fractions which were dried under a GeneVac HT-4X Series II system (Genevac Limited, UK). Selection of fractions for screening in the zebrafish epilepsy assay was performed by using the intensity of the ELSD signal detection. Using this approach, compound **1** (4.4 mg) was isolated from fraction B.

### NMR spectral data and quantification of inosine

#### Inosine (1)

White amorphous powder. ^1^H NMR (CD_3_OD, 500 MHz) δ 3.76 (2H, dd, *J* = 3.2; 12.3 Hz, H-5’), 3.88 (2H, dd, *J* = 3.2; 12.3 Hz, H-5’), 4.15 (1H, q, *J* = 3.2 Hz, H-4’), 4.33 (1H, dd, *J* = 3.5; 5.2 Hz, H-3’), 4.64 (1H, t, *J* = 5.7 Hz, H-2’), 6.03 (1H, d, *J* = 5.7 Hz, H-1’), 8.08 (1H, s, H-2), 8.35 (1H, s, H-8). ^13^C NMR (CD_3_OD, 126 MHz) δ 62.4 (C-5’), 87.3 (C-4’), 71.6 (C-3’), 75.3 (C-2’), 90.4 (C-1’), 124.7 (C-5), 140.8 (C-8), 146.6 (C-2), 148.0 (C-4), 157.1 (C-6). ESI-HRMS *m/z* 268.0704 [M + H]^-^ (calculated for C_10_H_12_N_4_O_5_, 268.0729).

#### Quantification of inosine

The quantification of (**1**) by ^1^H NMR was performed on a Bruker Avance III HD 600 MHz NMR spectrometer equipped with a QCI 5 mm Cryoprobe (Bruker BioSpin, Rheinstetten, Germany) (600.17 MHz). The ^1^H NMR spectra was recorded in a deuterated phosphate buffer solution (88.7 mM KH_2_PO_4_, pH 7 in D_2_O) containing 578.2 μM of TSP (3-(trimethylsilyl)propionic-2,2,3,3-d_4_ acid sodium). TSP was then used as the chemical shift reference (δ_H_ 0.0) and as the internal reference standard for quantification. The result was expressed as a percentage (w/w, dry weight).

### Zebrafish breeding and maintenance

Adult zebrafish (AB strain) were raised at 28°C on a 14/10 hours light/dark cycle according to standard aquaculture conditions [66]. Eggs were collected following natural spawning, sorted and reared in an embryo medium containing 0.3x of Danieau’s solution (1.5 mM HEPES, pH 7.6, 17.4 mM NaCl, 0.21 mM KCl, 0.12 mM MgSO_4_, and 0.18 mM Ca(NO_3_)_2_ under constant light conditions in an incubator set at 28°C until six or seven days post fertilization (dpf). Sorting of zebrafish embryos and larvae (based on phenotype and swimming behavior) and medium refreshment were performed daily until seven dpf. Upon completion of the zebrafish experiments reported here, all zebrafish larvae were immediately euthanized following regulatory guidelines. All zebrafish experiments performed in this study were approved by the Ethics Committee of the University of Luxembourg. In the experimental protocol reported here, PTZ-induced activity were used to evaluate the potential antiseizure activity of extracts/fractions/compounds within a defined time window. Based on the MTC of the tested samples, no appreciable levels of suffering were predicted nor observed that would require (a) monitoring for humane endpoints and/or (b) early euthanasia.

### Determination of the maximum tolerated concentration (MTC) in zebrafish larvae

To determine the range of appropriate concentrations tested in zebrafish for the evaluation of anticonvulsant activity before toxic signs, zebrafish larvae were incubated at different concentrations (10, 30, 100, 200 and 300 μg/mL) of samples dissolved in 1 mL of embryo medium (at a final DMSO concentration of 1%). The larvae were examined after 18 hours of incubation time and compared to the control group to detect the following signs of toxicity: absence of startle response to plate taps, presence of edema, loss of posture, paralysis and death. Thus, the MTC was defined as the highest concentration at which no signs of toxicity were observed in all replicates tested for one concentration.

### Evaluation of anticonvulsant activity in the zebrafish seizure models

Zebrafish larvae from six or seven dpf were monitored using the View Point Video Track System for Zebrafish™ (Version 2.3.1.0, ViewPoint, France) [5]. The system consisted in an infrared light source, a high resolution digital video camera to capture larval movements within a defined time period (30 min in our experimental set-up) and the software to analyze larval locomotor activity. The highest concentrations of the samples tested correspond to the previously determined MTC values. Zebrafish larvae were placed in a flat bottom 96-well plate (Falcon^®^, USA) (1 larvae/well, at least 3 replicates for each concentration tested, e.g. 10, 30, 50 and 100, 200, 300 μg/mL) and incubated at 28°C for 18 hours (as performed in [5]). Larvae medium added to DMSO (1% w/v) was used as the vehicle control. Pentylenetetrazole (PTZ) added to the vehicle at 20 mM served as positive control. PTZ added to the medium elicits distinct seizure-like behaviors characterized by greatly increased locomotor activity (Stage I) and ‘‘whirlpool-like” circling swim behavior (Stage II). Thus, normal locomotor activity and seizures could have been differentiated by seeing a significant increased ratio between movement with vehicle only and vehicle with PTZ. Moreover, each tracking experiment has been videotaped as part of this research and fish seizures has been determined based on the ‘‘whirlpool-like” swimming behavior (Stage II seizure) [67]. The toxicity of methanol in 6–7 dpf larvae have been studied by Maes et al. who assessed a 2% tolerance to this solvent [68]. Movements of fish larvae were recorded for 30 min with an automated video-tracking from ViewPoint^®^. Then, PTZ solution was added in each well (except the negative control). Video-tracking of larval movements commenced exactly 5 min after addition of PTZ solution to the wells and was recorded again for 30 min. Data were saved by the software (Viewpoint^®^) each 5 min as an average value until 30 min of total larval movements. Results were interpreted in actinteg and time units [5]. Actinteg unit is determined by the sum of pixel shifts detected for each larva during the reported time period (e.g. 30 min).

### Statistical analysis

Data were expressed as mean ± SEM (n ≥ 3). Statistical differences among the data were assessed by a one-way ANOVA with Dunett’s test to compare samples with positive control, with *P* values of <0.05 (*), <0.01 (**), <0.001 (***), <0.0001 (****). Analyses were performed using the statistical software Prism v7.02 (GraphPad Software).

## Conclusions

This study describes a zebrafish-based screening for neuroactivity and antiseizure activity of *Skeletonema marinoi*, a marine diatom which has previously shown antimicrobial, anticancer and antioxidant activities [28, 69]. The bioassay-guided investigation of its methanolic extract led to the isolation of the bioactive nucleoside inosine, which significantly reduced PTZ-induced locomotor activity in a zebrafish epilepsy assay. Inosine is reported here for the first time in *S*. *marinoi*. We also report 11 other metabolites described in the UHPLC-TOF-HRMS profiling, thereby further documenting the chemical composition of *S*. *marinoi*. This study demonstrates the potential of marine diatoms as a source of anticonvulsant compounds.

## Supporting information

S1 Fig Larval locomotor activity (expressed in actinteg units) of the 13 richest fractions from the ELSD chromatogram after fractionation by preparative chromatography. (A) Fractions A to D. (B) Fractions E to I. (C) Fractions J to M. Fractions are tested at 10, 30 and 100 μg/mL and data are represented as actinteg mean ± SEM (n ≥ 3). PTZ was used at 20 mM as the proconvulsant agent. Controls are described as “-” for the vehicle control and “+” for the positive control.
(PDF)

S2 Fig Correlations of inosine with COSY (blue bonds) and HMBC (black arrows) (500 MHz, CD_3_OD). (PDF)

S3 Fig ^1^H NMR (500 MHz, CD_3_OD) spectrum of inosine. (PDF)

S4 Fig COSY NMR (500 MHz, CD_3_OD) spectrum of inosine. (PDF)

S5 Fig HSQC NMR (500 MHz, CD_3_OD) spectrum of inosine. (PDF)

S6 Fig HMBC NMR (500 MHz, CD_3_OD) spectrum of inosine. (PDF)

S1 Table ^1^H and ^13^C NMR assignments of inosine (500 MHz, CD_3_OD). (PDF)
