# Supplementary material for: Zebrafish-based identification of the antiseizure nucleoside inosine from the marine diatom Skeletonema marinoi
Source: PLoS One. 2018 Apr 24;13(4):e0196195. doi: 10.1371/journal.pone.0196195 (PMC5916873; doi:10.1371/journal.pone.0196195)

S1 Figure. Larval locomotor activity (expressed in actinteg units) of the 13 richest fractions from the ELSD chromatogram after fractionation by preparative chromatography.

(A) Fractions A to D. (B) Fractions E to I. (C) Fractions J to M. Fractions are tested at 10, 30 and 100 µg/mL and data are represented as actinteg mean ± SEM (n ≥ 3). PTZ was used at 20 mM as the proconvulsant agent. Controls are described as "-" for the vehicle control and "+" for the positive control.

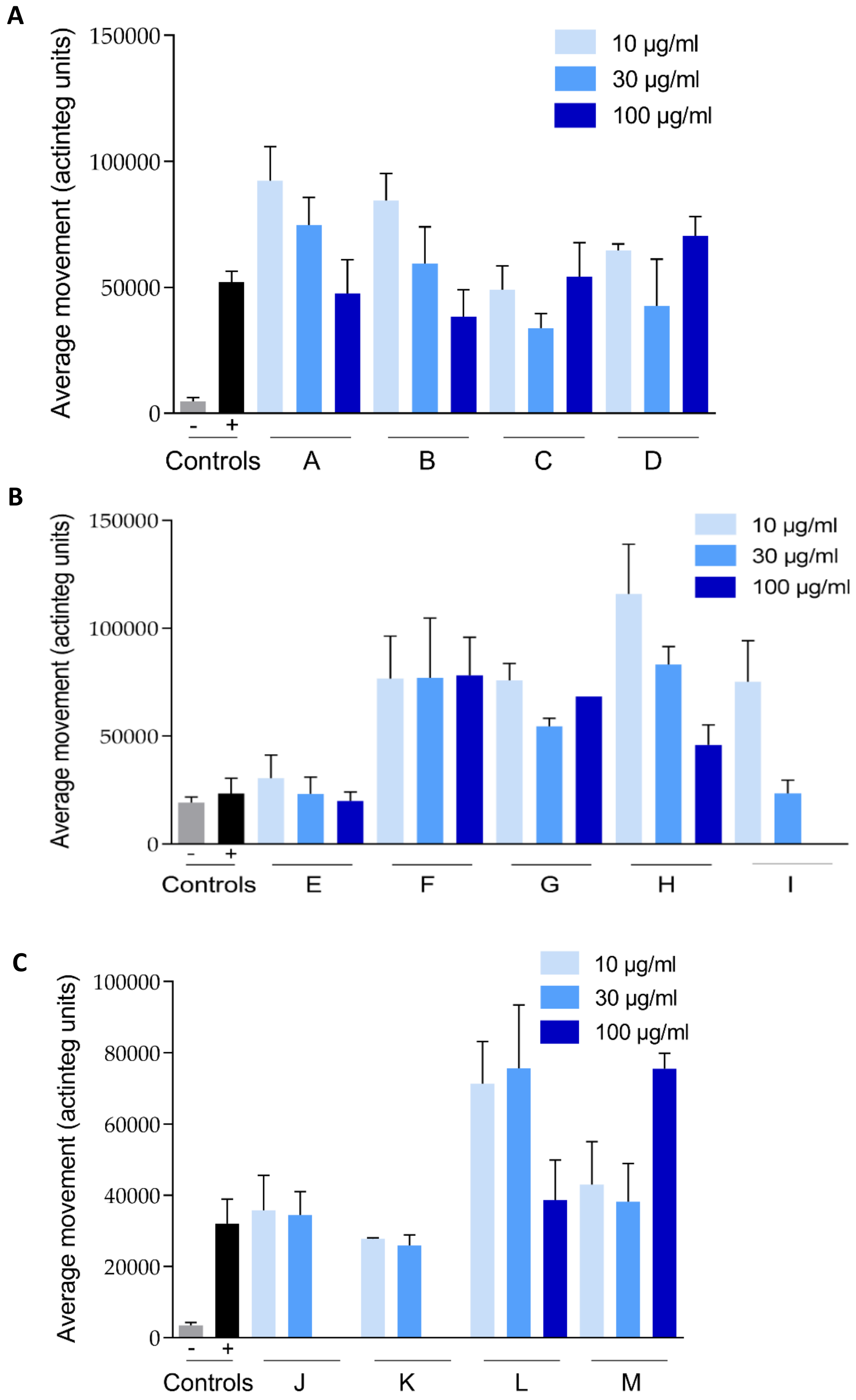

Supplement: S1 Fig — (A) Fractions A to D. (B) Fractions E to I. (C) Fractions J to M. Fractions are tested at 10, 30 and 100 μg/mL and data are represented as actinteg mean ± SEM (n ≥ 3). PTZ was used at 20 mM as the proconvulsant agent. Controls are described as “-” for the vehicle control and “+” for the positive control. (PDF) [file pone.0196195.s001.pdf]
