# Supplementary figures and images for: Zebrafish-based identification of the antiseizure nucleoside inosine from the marine diatom Skeletonema marinoi
Source: PLoS One. 2018 Apr 24;13(4):e0196195. doi: 10.1371/journal.pone.0196195 (PMC5916873; doi:10.1371/journal.pone.0196195)

S2 Figure. Correlations of inosine with COSY (blue bonds) and HMBC (black arrows) (500 MHz, CD<sub>3</sub>OD)

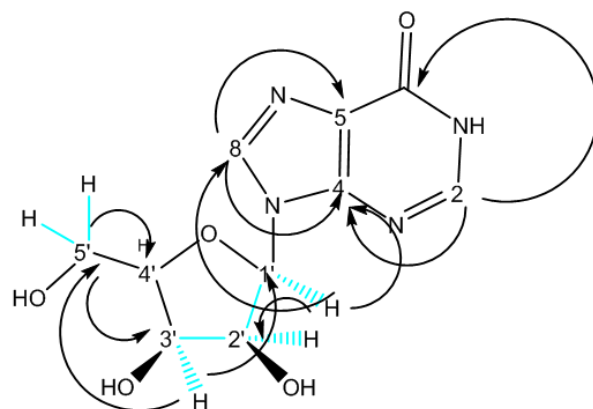

Supplement: S2 Fig — (PDF) [file pone.0196195.s002.pdf]

S3 Figure.  $^1\text{H}$  NMR (500 MHz,  $\text{CD}_3\text{OD}$ ) spectrum of inosine

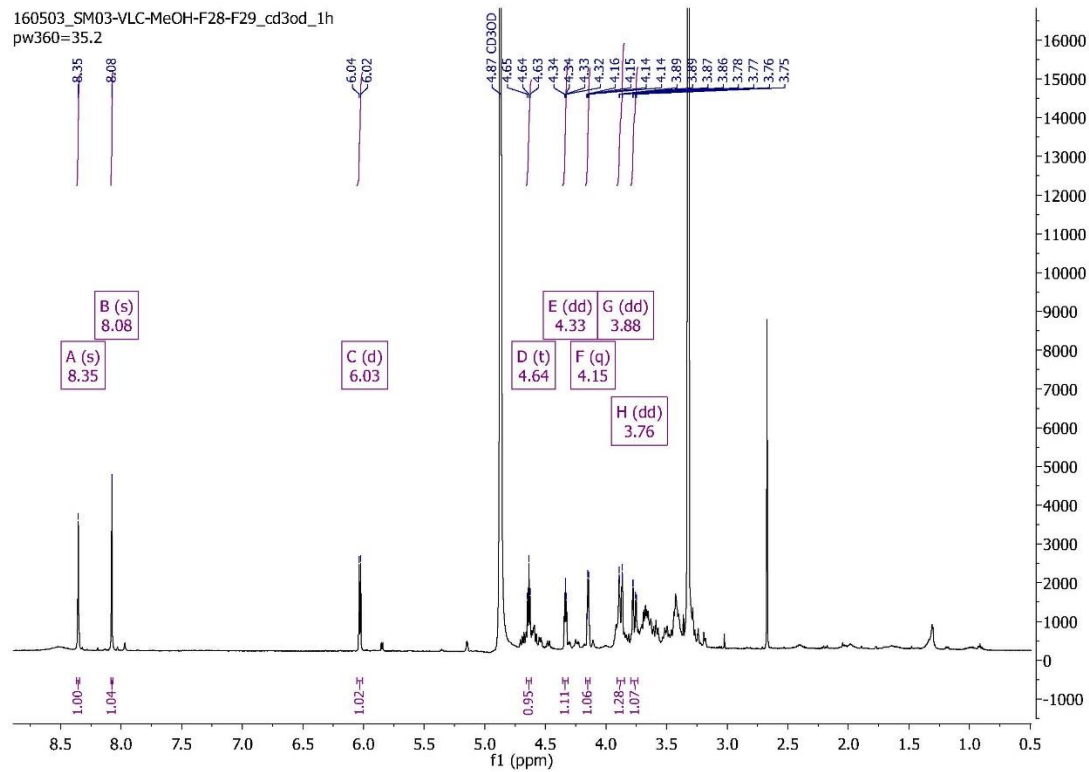

Supplement: S3 Fig — (PDF) [file pone.0196195.s003.pdf]

S4 Figure. COSY NMR (500 MHz, CD<sub>3</sub>OD) spectrum of inosine

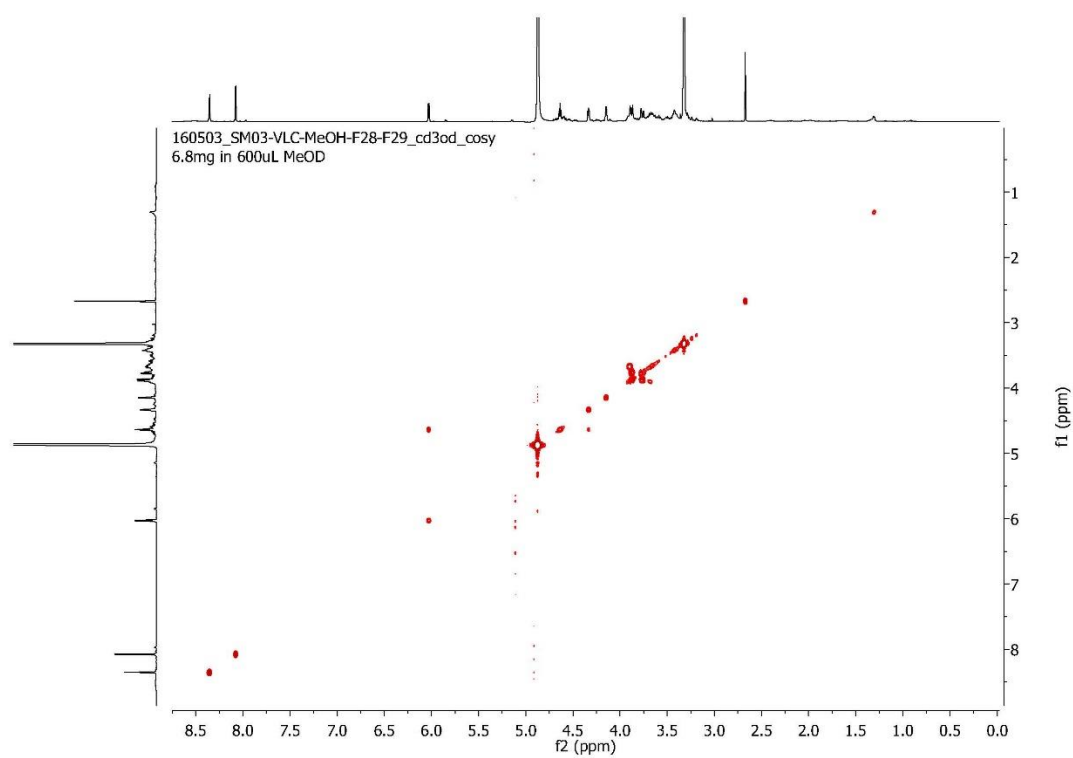

Supplement: S4 Fig — (PDF) [file pone.0196195.s004.pdf]

S5 Figure. HSQC NMR (500 MHz, CD<sub>3</sub>OD) spectrum of inosine

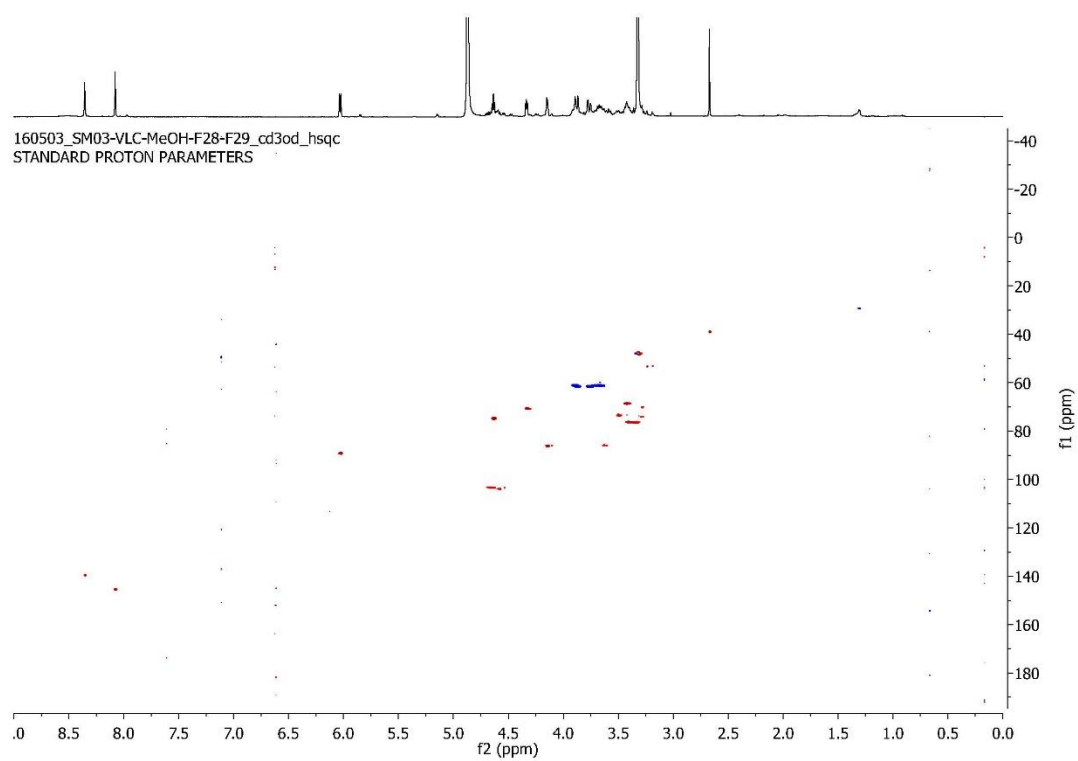

Supplement: S5 Fig — (PDF) [file pone.0196195.s005.pdf]

**S6 Figure. HMBC NMR (500 MHz, CD<sub>3</sub>OD) spectrum of inosine**

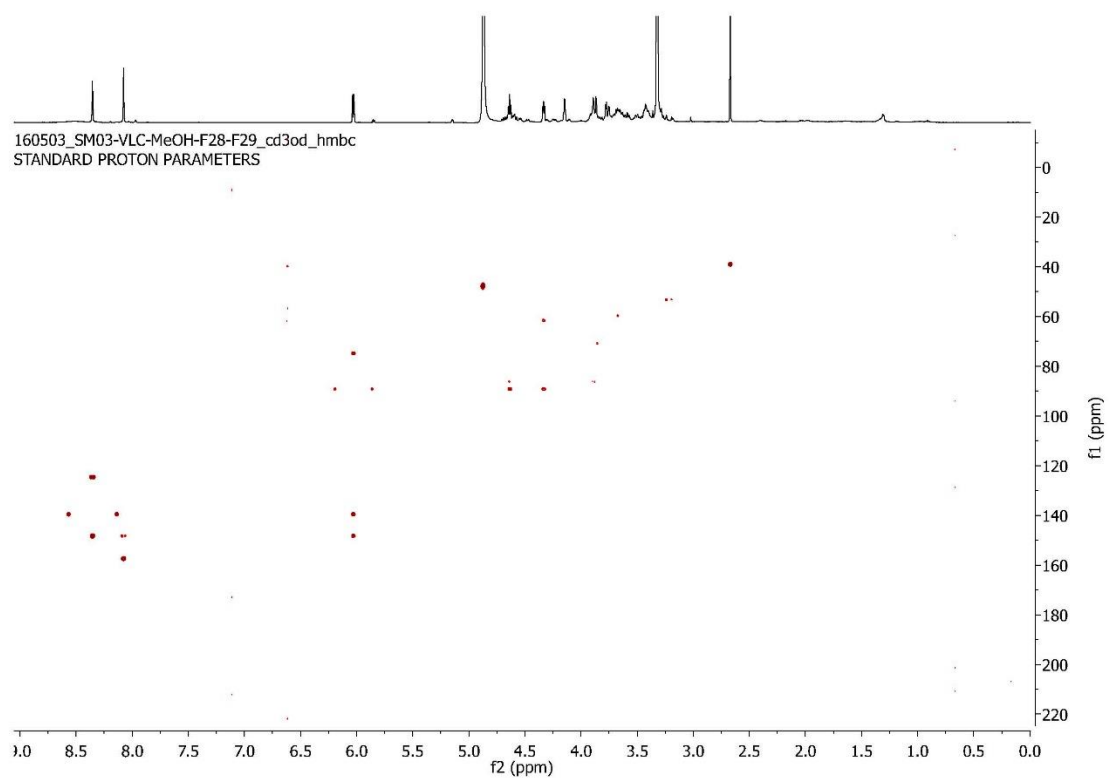

Supplement: S6 Fig — (PDF) [file pone.0196195.s006.pdf]
