# Supplementary material for: Zebrafish-based identification of the antiseizure nucleoside inosine from the marine diatom Skeletonema marinoi
Source: PLoS One. 2018 Apr 24;13(4):e0196195. doi: 10.1371/journal.pone.0196195 (PMC5916873; doi:10.1371/journal.pone.0196195)
Supplement: S1 Table — (PDF) [file pone.0196195.s007.pdf]

S1 Table. ^{1}H and ^{13}C NMR assignments of inosine (500 MHz, CD_3OD)

	^{1}H [(δ, mult, J (Hz)]	^{13}C
2	8.08 s	146.6
4		148.0
5		124.7
6		157.1
8	8.35 s	140.8
1’	6.03 d (5.7)	90.4
2’	4.64 t (5.7)	75.3
3’	4.33 dd (3.5, 5.2)	71.6
4’	4.15 q (3.2)	87.3
5a’	3.88 dd (3.2, 12.3)	62.4
5b’	3.76 dd (3.2, 12.3)	
